# Downregulation of circ-YES1 suppresses NSCLC migration and proliferation through the miR-142-3p–HMGB1 axis

**DOI:** 10.1186/s12931-023-02378-6

**Published:** 2023-04-03

**Authors:** Mingming Jin, Yan Wang, Dawei Zhou, Wanchao Liu, Ruodong Han, Yongbin Chi

**Affiliations:** 1 Shanghai Key Laboratory of Molecular Imaging, Jiading District Central Hospital Affiliated Shanghai University of Medicine and Health Sciences, Shanghai, 201318 People’s Republic of China; 2 Department of Clinical Lab, Shanghai Health Commission Key Lab of Artificial Intelligence (AI)-Based Management of Inflammation and Chronic Diseases, Sino-French Cooperative Central Lab, Shanghai Pudong Gongli Hospital, Secondary Military Medical University, Shanghai, 200135 People’s Republic of China; 3 Department of Ultrasonics, Shanghai Baoshan Hospital of Integrated Traditional Chinese and Western Medicine, Shanghai, 201901 People’s Republic of China; 4 Department of Clinical Laboratory, Baoshan District Integrative Medicine Hospital, Shanghai, Shanghai 201901 People’s Republic of China; 5 Department of Critical Care Medicine, The Affiliated Bozhou Hospital of Anhui Medical University, Bozhou, 236800 People’s Republic of China

**Keywords:** Non-small cell lung cancer, Circ-YES1, miR-142-3p, High mobility group protein B1, Proliferation, Migration

## Abstract

**Background:**

Circular RNAs (circRNAs) are a new family of abundant regulatory RNAs with roles in various types of cancer. While the hsa_circ_0046701 (circ-YES1) function in non-small cell lung cancer (NSCLC) is unclear.

**Methods:**

Circ-YES1 expression in normal pulmonary epithelial and NSCLC cells was examined. The small interfering RNA for circ-YES1 was prepared, cell proliferation and migration were assessed. Tumorigenesis in nude mice was assayed to validate the role of circ-YES1. Bioinformatics analyses and luciferase reporter assays were utilized to identify downstream targets of circ-YES1.

**Results:**

Compared to normal pulmonary epithelial cells, the circ-YES1 expression increased in NSCLC cells, and cell proliferation and migration were suppressed after circ-YES1 knockdown. Both high mobility group protein B1 (HMGB1) and miR-142-3p were found to be downstream targets of circ-YES1, and miR-142-3p inhibition and HMGB1 overexpression reversed the effects of circ-YES1 knockdown on cell proliferation and migration. Similarly, HMGB1 overexpression reversed the miR-142-3p overexpression effects on these two processes. The imaging experiment results revealed that circ-YES1 knockdown impeded tumor development and metastasis in a nude mouse xenograft model.

**Conclusion:**

Taken together, our results show that circ-YES1 promotes tumor development through the miR-142-3p–HMGB1 axis and support the development of circ-YES1 probability as a new therapeutic NSCLC target.

## Background

Lung cancer is an extremely common and fatal malignancy worldwide. Presently, the lung cancer 5-year survival rate is < 15% [1]. More than four-fifths of all lung cancers are characterized as non-small cell lung cancer (NSCLC), which is the most common subtype. Unfortunately, NSCLC is often diagnosed in advanced stages of the trait [1, 2]. As such, further studies are needed to improve our understanding of NSCLC in order to identify new therapeutic targets and diagnostic biomarkers.

Circular RNAs (circRNAs) are novel and abundant non-coding RNAs that are highly stable due to their covalent closed-loop structure [3]. In general, the expression of circRNAs is species-, tissue-, and cell-specific [4, 5]. The majority of circRNAs are < 1500 nucleotides in length, with an average length of ~ 500 nucleotides [6, 7]. Several circRNAs have been reported to associate with the recurrence and prognosis of NSCLC [8–10]. For instance, circ-0014130 has been reported to participate in cell proliferation and apoptosis via the miR-142-5p–IGF-1 axis in NSCLC [11], whereas hsa_circ_0002483 has been demonstrated to impede tumor progression and enhance the sensitivity of cells to Taxol by targeting miR-182-5p [12]. In yet another in vitro and in vivo study, hsa_circ_0046701 (circ-YES1) was significantly up-regulated in glioma, and circ-YES1 knockdown was found to inhibit cell invasion and proliferation [13]. Here, we report abnormal circ-YES1 expression in NSCLC and examine the circ-YES1 roles in NSCLC progression and development.

## Methods

### Ethics statement

We procured four-week-old BALB/c nude female mice weighing 15 ~ 20 g from SLARC (Shanghai, China). Shanghai University of Medicine and Health Sciences Ethics Committee approved animal experiments (No.2020-GZR-18-340406198707142817).

### Cell culture

We purchased human pulmonary epithelial cells (BEAS-2B) and NSCLC cells (H1650, A549, and PC-9) from American Type Culture Collection (Manassas, VA, USA). We cultured BEAS-2B cells in RPMI-1640 medium (Gibco, Grand Island, NY, USA) supplemented with 10% (v/v) fetal bovine serum (FBS) in humidified atmosphere of 5% CO_2_ at 37 °C. NSCLC cells (H1650, A549, and PC-9) were Dulbecco’s modified Eagle media (DMEM) supplemented with 10% (v/v) FBS in humidified atmosphere of 5% CO_2_ at 37 °C.

### RNA interference or overexpression

The miR-142-3p mimics, miR-142-3p inhibitor, circ-YES1 silence vector (si-circ-YES1), HMGB1 overexpression vector (HMGB1) (constructed the HMGB1 cDNA sequence into the cDNA3.1 vector) were obtained from RiboBio (Guangzhou, China), and we conducted transfection via Lipofectamine 2000 (Thermo Fisher Scientific).

### Cell proliferation and clone formation assays

We assessed cell proliferation through Cell Counting Kit-8 (CCK-8) assay (Invitrogen, Carlsbad, CA, USA). Differently-treated cells (2 × 10^3^/well) were added in triplicate to wells of 96-well plates and cultured for 0, 24, 48, 72, and 96 h. We examined cell proliferation after each time point. Colony formation was assessed in differently-treated cells (2 × 10^3^/well) cultured in DMEM supplemented with 10% (v/v) FBS in wells of 6-well plates for 10 d. Colonies were fixed, stained, counted, and photographed.

### Cell migration assays

We performed cell migration assays in Transwell chambers (membrane pore size, 8 µm; BD Biosciences, Franklin Lakes, NJ, USA) housed within 24-well plates. We added differently-treated cells (1 × 10^5^/unit) in 200 µL of serum-free media to upper chamber. We filled lower chamber with 500 µL of complete media, which served as the chemoattractant. Cells that had migrated through membrane pores after 1 d were fixed with 4% (w/v) paraformaldehyde for 0.5 h and stained with Crystal violet for 10 min.

### Amplification of target genes by quantitative polymerase chain reactions (qPCR)

We extracted total RNA with TRIzol reagent (Invitrogen), and synthesized cDNA through the pTRUEscript First Strand cDNA Synthesis kit (Aidlab, Beijing, China). To amplify target genes, quantitative polymerase chain reactions (qPCR) were carried out in the presence of gene-specific primers and 2 × SYBR Green qPCR Mix (Invitrogen) in an ABI 7900HT Fast Real-Time PCR system (Thermo Fisher Scientific, Waltham, MA, USA). The primers were as follows: 5′-GAAATTGGTGAAACAC-3′ (forward) and 5′-GCTTCATAATCATATAAGGC-3′ (reverse) for circ-YES1; 5′-GTCGTATCCAGTGCAGGG-3′ (forward) and 5′-CGACGTGTAGTGTTTCCTA-3′ (reverse) for miR-142-3p; 5′-AGGCTGACAAGGCTCGTTATG-3′ (forward) and 5′-TGTCATCCGCAGCAGTGTTG-3′ (reverse) for high mobility group protein B1 (HMGB1); 5′-CTCGCTTCGGCAGCACA-3′ (forward) and 5′-AACGCTTCATTTGCGT-3′ (reverse) for U6; and 5′-AATCCCATCACCATCTTCC-3′ (forward) and 5′-CATCACGCCACAGTTTCC-3′ (reverse) for GAPDH. The miR-142-3p primer for the reverse transcription reaction was 5′-GTCGTATCCAGTGCAGGGTCCGAGGTATTCGCACTGGATACGA CTCCATAAA-3′. We calculated fold-change in expression by 2^−ΔΔCT^ method. The expression levels of circ-YES1 and HMGB1 were normalized to that of GAPDH, whereas we normalized the miR-142-3p expression level to that of U6.

### Tumor xenograft formation and metastasis assays

H1650 cells (5 × 10^6^) transfected with circ-YES1 siRNA or negative control (NC) siRNA were injected into female BALB/c nude mice (*n* = 6) right flank. We measured tumors every five days using vernier calipers, and calculated the tumor volume as = 0.5 × length × width^2^. Mice were euthanized after 30 d, and tumor specimens were used in Ki-67 immunohistochemistry experiments.

H1650 cells (2 × 10^5^) transfected with circ-YES1 siRNA or empty plasmids were intravenously injected into the tails of mice. Metastasis was assessed after 1 month by intravenously injecting luciferin (150 mg/kg body weight) and imaging the whole body. The numbers of metastatic foci in lung tissues were caculation according to the HE staining.

### Dual luciferase reporter assays

We prepared reporter plasmids through inserting circ-YES1 or the 3′ untranslated region (UTR) of HMGB1 into the pmirGLO vector (Promega, Madison, WI, USA). Subsequently, reporter plasmids were individually cotransfected with miR-142-3p inhibitor into human embryonic kidney (HEK) 239T cells with Lipofectamine 2000 transfection reagent. After 2 d of incubation, firefly and Renilla luciferase activity was measured with Dual Luciferase Reporter Assay system (Promega), following manufacturer’s instructions.

### Statistical analyses

Differences between groups were assessed using one-way ANOVA method with Tukey’s post hoc test. Results were presented as the means ± SEM. P-values < 0.05 were considered to indicate statistically significant differences. Statistical analyses were performed using GraphPad Prism 5.02 software (GraphPad Inc.).

## Results

### Circ-YES1 expression is increased in NSCLC cells and circ-YES1 knockdown suppresses cell proliferation and migration

**Fig. 1:**
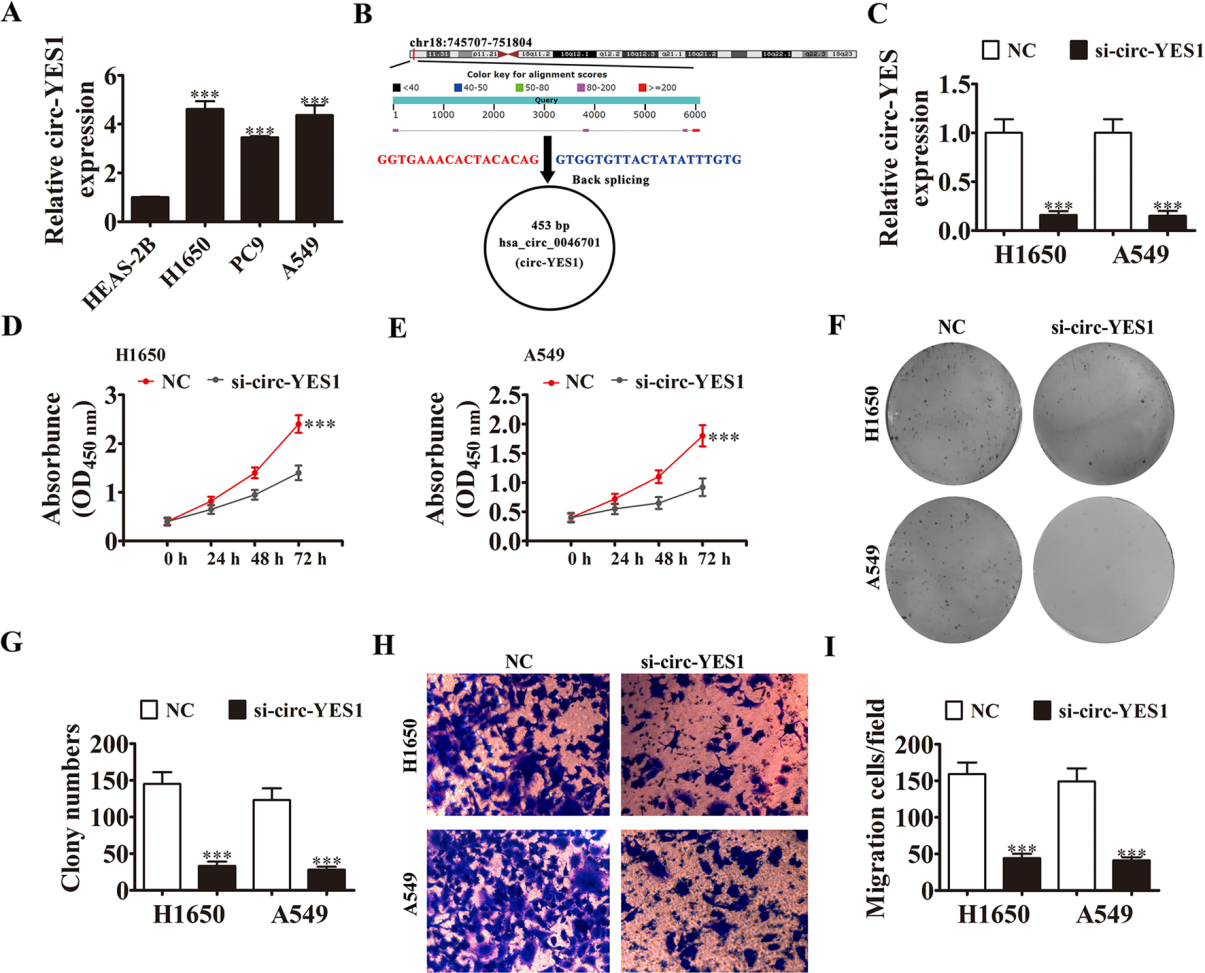
Expression of circ-YES1 was increased in NSCLC cells and circ-YES1 knockdown inhibited cell proliferation and migration. **A** Expression of circ-YES1 in H1650, PC-9, A549, and HEAS-2B cells by qPCR. n = 3. Data are presented as means ± SD. ***P < 0.001 vs. HEAS-2B. **B** Genomic loci of the *YES1* gene and circ-YES1. The black arrow indicates back-splicing. **C** Expression of circ-YES1 after transfection of cells with circ-YES1 siRNA (si-circ-YES1) or negative control siRNA (NC). n = 3. Data are presented as means ± SD. ***P < 0.001 vs. NC. **D** and **E** H1650 (**D**) and A549 (**E**) cell proliferation as assessed by CKK-8 assays. Data are presented as means ± SD. ***P < 0.001 vs. NC. n = 3. **F** and **G** H1650 and A549 cell proliferation as assessed by clone formation assays. n = 3. Data are presented as means ± SD. ***P < 0.001 vs. NC. **H** and **I** H1650 and A549 cell migration as assessed by Transwell assays. n = 3. Data are presented as means ± SD. ***P < 0.001 vs. NC

The hsa_circ_0046701 (circ-YES1) expression level was higher in H1650 and A549 cells than that in BEAS-2B cells (Fig. 1A); therefore, all subsequent experiments were performed in H1650 and A549 cells. In bioinformatics analysis, hsa_circ_0046701 was found to be located on chromosome chr18:745707-751804, and hsa_circ_0046701 was derived from exon 4 of the *YES1* gene, with a length of 453 bp. Therefore, hsa_circ_0046701 was designated as circ-YES1 (Fig. 1B). In qPCR analysis, the circ-YES1 level was significantly lower in both H1650 and A549 cells than that in BEAS-2B cells (Fig. 1C). Furthermore, circ-YES1 knockdown suppressed cell proliferation as observed in CCK-8 (Fig. 1D, E) and clone formation (Fig. 1F, G) assays. In addition, circ-YES1 knockdown reduced the migration of H1650 and A549 cells (Fig. 1H, I), indicating that circ-YES1 has an important role in this process.

### HMGB1 and miR-142-3p are circ-YES1 downstream targets, and miR-142-3p inhibition and HMGB1 overexpression can reverse the circ-YES1 knockdown effects on cell proliferation and migration

**Fig. 2:**
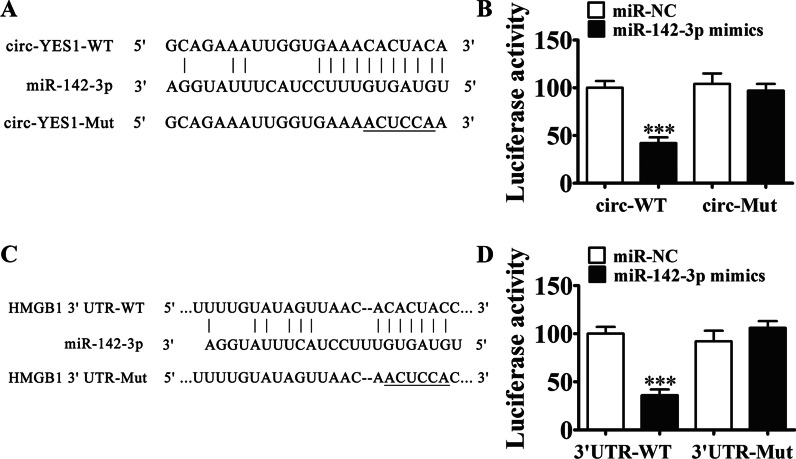
HMGB1 and miR-142-3p are downstream targets of circ-YES1. **A** The binding sites of miR-142-3p in circ-YES1 are shown. The mutated circ-YES1 is also shown. **B** Relative luciferase activity was measured 48 h after transfection of HEK293T cells with miR-142-3p mimic/negative control (NC) or circ-YES1 wild type (wild)/mutant (Mut). n = 3. Data are presented as means ± SD. ***P < 0.001. **C** The binding sites of miR-142-3p with the 3′ UTR of circ-YES1 are shown. The mutated 3′ UTR of circ-YES1 is also shown. **D** Relative luciferase activity was measured 48 h after transfection of HEK293T cells with miR-142-3p mimic/NC or 3′ UTR-circ-YES1 wild/Mut. n = 3. Data are presented as means ± SD. ***P < 0.001

Bioinformatics analysis (http://starbase.sysu.edu.cn/) revealed that miR-142-3p was circ-YES1 downstream target, which prompted us to generate wild-type (WT) and mutant miR-142-3p luciferase reporter plasmids (Fig. 2A). We found that luciferase activity decreased after cotransfection with WT circ-YES1 and miR-142-3p mimics (Fig. 2B), inferring that circ-YES1 directly interacts with miR-142-3p. Bioinformatics analysis (http://starbase.sysu.edu.cn/) also revealed that HMGB1 was miR-142-3p downstream target (Fig. 2C), and the results of luciferase reporter assays confirmed that miR-142-3p binds to the HMGB1 3' UTR (Fig. 2D).

**Fig. 3:**
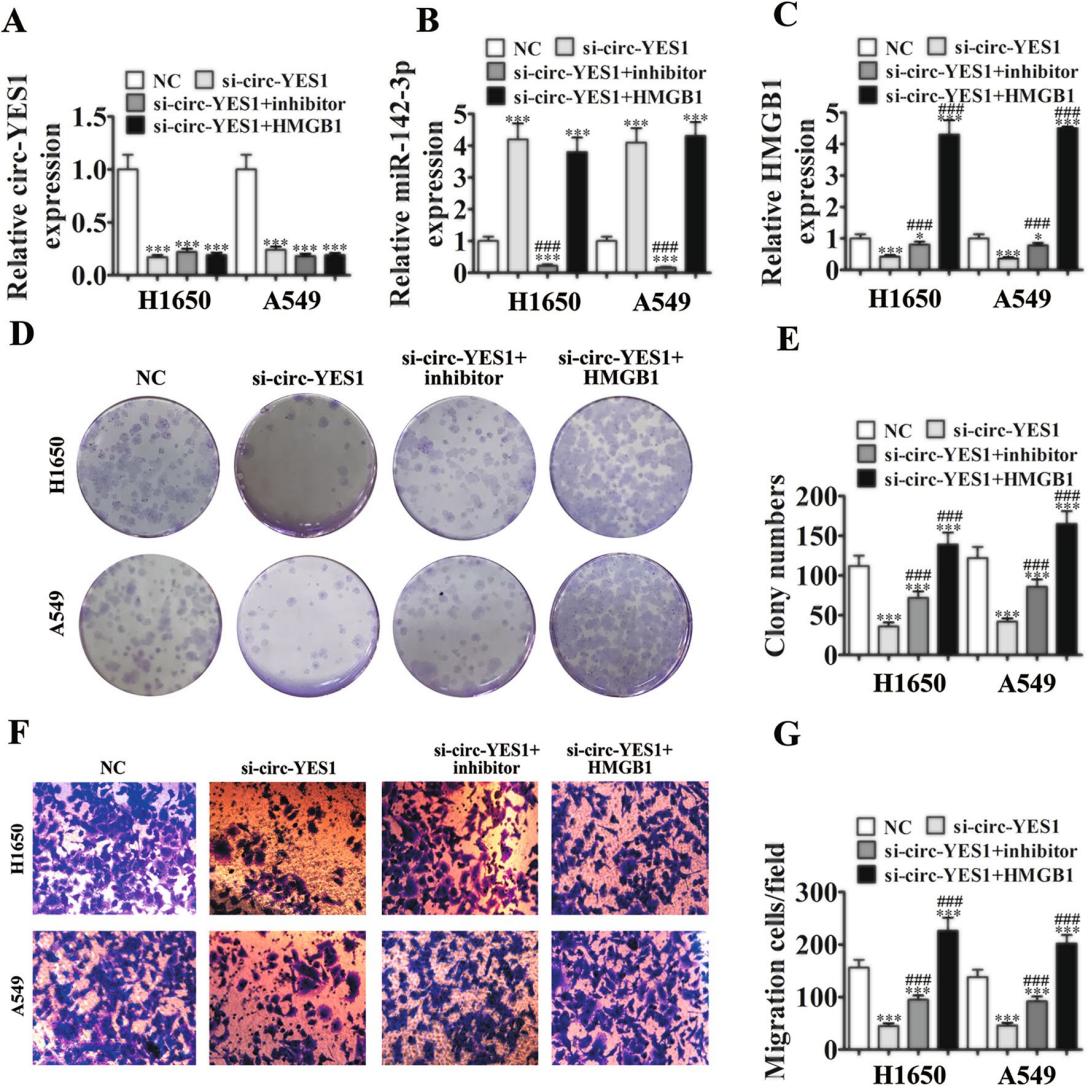
MiR-142-3p knockdown and HMGB1 overexpression reversed the effects of circ-YES1 knockdown on the proliferation and migration of H1650 and A549 cells. **A**–**C** Expression of circ-YES1 (**A**), miR-142-3p (**B**), and HMGB1 (**C**) by qPCR. n = 3. Data are presented as means ± SD. *P < 0.05; ***P < 0.001 vs NC; ^###^*P* < 0.001 vs si-circ-YES1. **D** and **E** H1650 and A549 cell proliferation as assessed by clone formation assays. Data are presented as means ± SD. ***P < 0.001 vs. NC; ^###^*P* < 0.001 vs si-circ-YES1. **F** and **G** H1650 and A549 cell migration as assessed by Transwell assays. n = 3. Data are presented as means ± SD. ***P < 0.001 vs. NC; ^###^*P* < 0.001 vs si-circ-YES1

We transfected H1650 and A549 cells with circ-YES1 siRNA, miR-142-3p inhibitor, or the HMGB1-overexpressing plasmid alone or in combination. The results of qPCR advised that circ-YES1 was successfully silenced. Furthermore, circ-YES1 knockdown decreased the miR-142-3p expression level, and HMGB1 overexpression cannot recover the circ-YES1 expression (Fig. 3A), indicating that miR-142-3p and HMGB1 are downstream targets of circ-YES1. Furthermore, circ-YES1 knockdown increased the miR-142-3p expression level, and HMGB1 overexpression could not down-regulate miR-142-3p, even after circ-YES1 knockdown. Similarly, HMGB1 overexpression could not down-regulate miR-142-3p, even after circ-YES1 knockdown (Fig. 3B), indicating that HMGB1 is a miR-142-3p downstream target. Circ-YES1 knockdown decreased the HMGB1 expression level; however, decreased miR-142-3p expression partially rescued HMGB1 expression (Fig. 3C), confirming that HMGB1 was a miR-142-3p downstream target.

In clone formation analysis, circ-YES1 knockdown inhibited the proliferation of both H1650 and A549 cells. Interestingly, miR-142-3p knockdown and HMGB1 overexpression could reverse the circ-YES1 knockdown effects on proliferation (Fig. 3D, E) and migration (Fig. 3F, G) in both cell types.

### HMGB1 overexpression can restore cell proliferation and migration after miR-142-3p overexpression

**Fig. 4:**
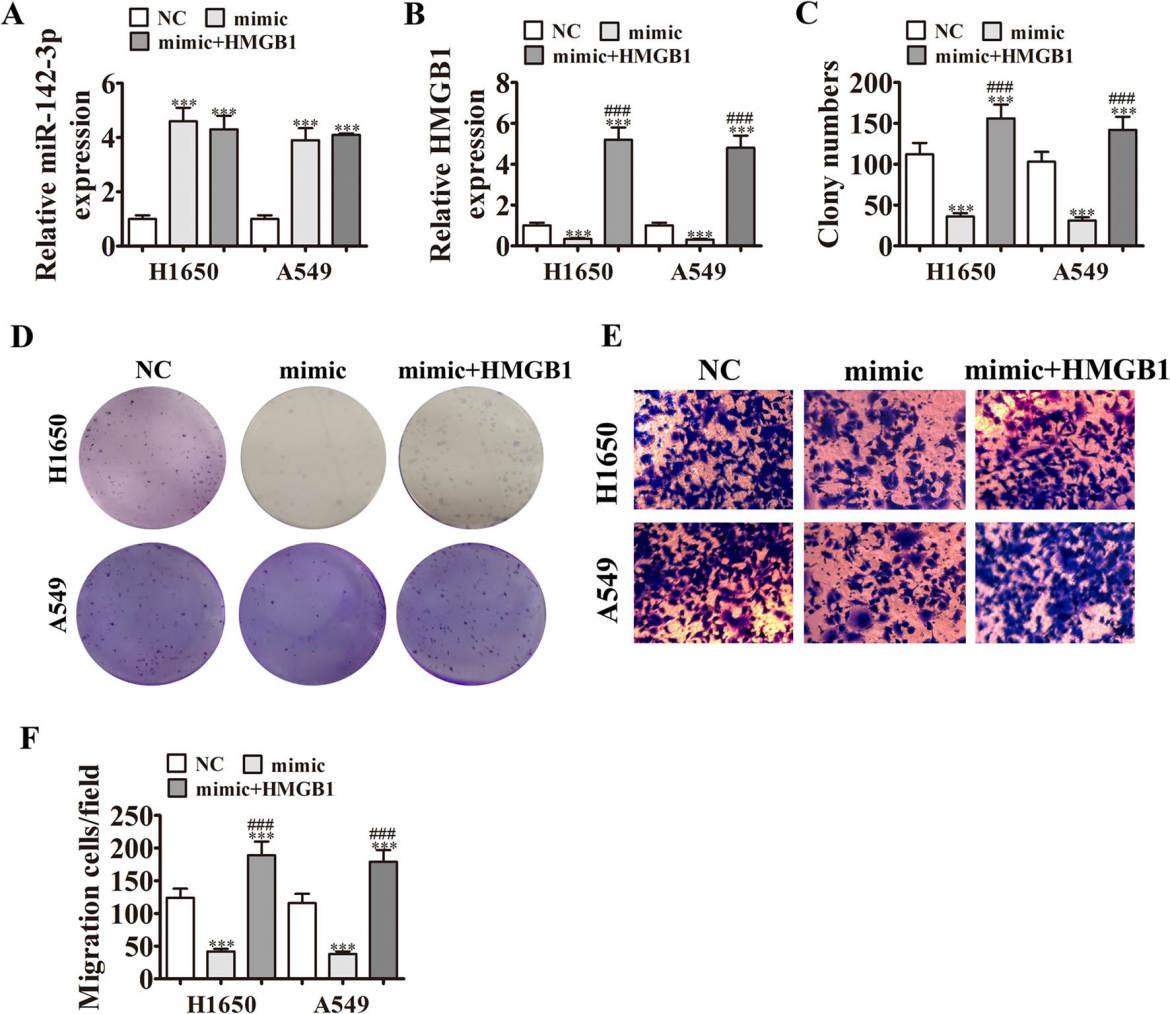
HMGB1 overexpression reversed the effects of miR-142-3p overexpression on the proliferation and migration of H1650 and A549 cells. **A** and **B** Expression of miR-142-3p (**A**) and HMGB1 (**B**) by qPCR. n = 3. Data are presented as means ± SD. ***P < 0.001 vs NC; ^###^*P* < 0.001 vs inhibitor. **C** and **D** H1650 and A549 cell proliferation as assessed by clone formation assays. n = 3. Data are presented as means ± SD. ***P < 0.001 vs. NC; ^###^*P* < 0.001 vs mimic. **E** and **F** H1650 and A549 cell migration as assessed by Transwell assays. n = 3. Data are presented as means ± SD. ***P < 0.001 vs. NC; ^###^*P* < 0.001 vs inhibitor

The relationship between HMGB1 and miR-142-3p was further examined. In qPCR analysis, miR-142-3p expression increased significantly after transfected with miR-142-3p mimic. HMGB1 overexpression could not down-regulate miR-142-3p (Fig. 4A), although miR-142-3p overexpression could down-regulate HMGB1. As the heterogenic HMGB1 mRNA is reported to lack a 3' UTR, miR-142-3p cannot affect the HMGB1 mRNA level, which explains why HMGB1 expression was increased after HMGB1 overexpression (Fig. 4B). In clone formation analysis, HMGB1 overexpression rescued the proliferation ability of both cell types under miR-142-3p upregulation condition (Fig. 4C, D). Furthermore, miR-142-3p overexpression inhibited cell migration, although HMGB1 overexpression could reverse the miR-142-3p overexpression effects (Fig. 4E, F), indicating that HMGB1 can restore the proliferation and migration of H1650 and A549 cells.

### Knockdown of circ-YES1 suppresses tumor development and metastasis in a nude mouse xenograft model

**Fig. 5:**
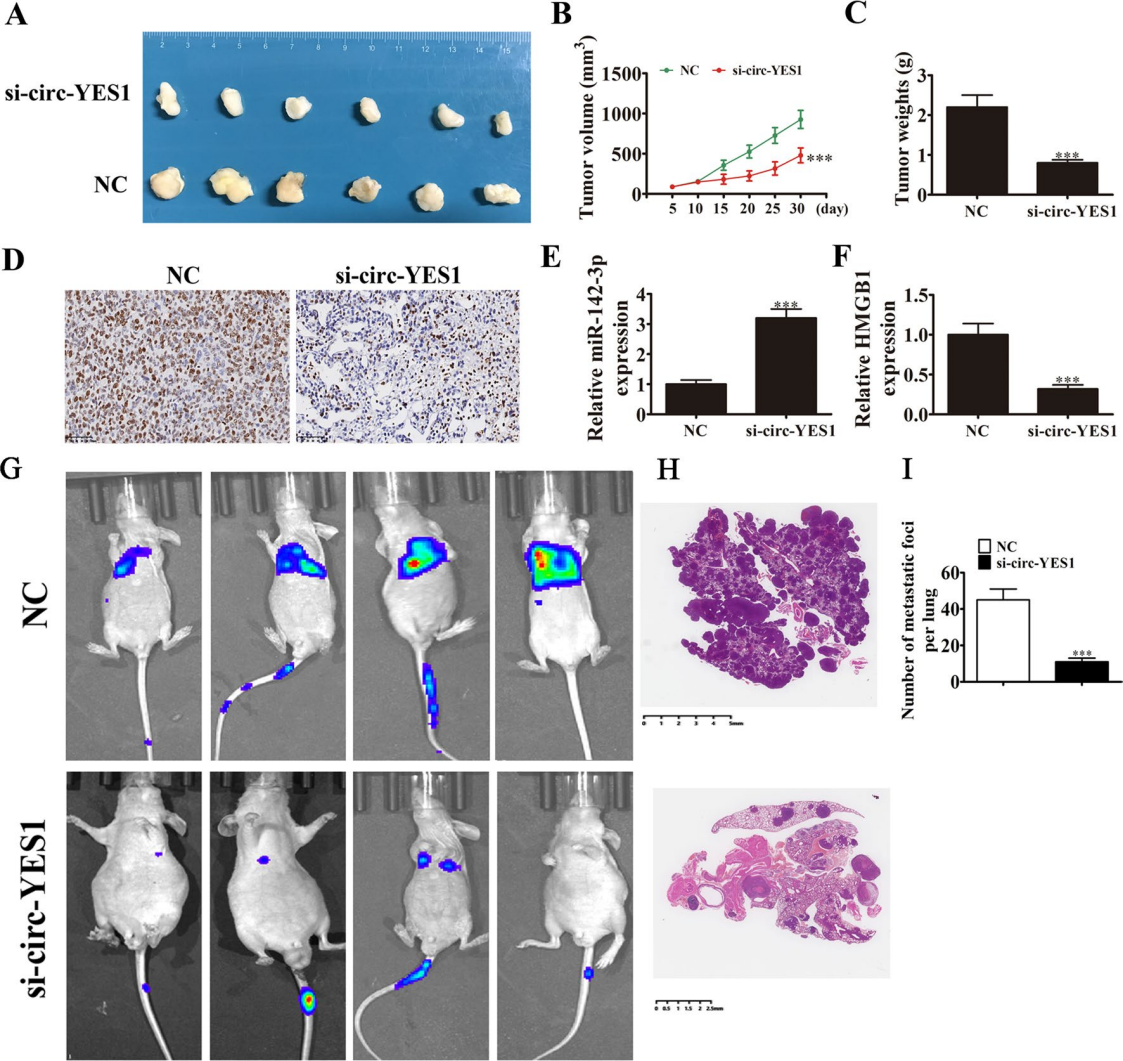
Circ-YES1 knockdown impedes tumor development and metastasis in a nude mouse xenograft model. **A** Representative images of nude mouse xenografts after injection of H1650 cells. n = 6. **B** Tumors were measured every 5 days. n = 6. Data are presented as means ± SD. ***, *P* < 0.001 vs. NC. **C** Tumor weight was measured 30 d after grafting. Data are presented as means ± SD. ***, *P* < 0.001 vs. NC. **D** Immunohistochemical staining of Ki-67 in tumor specimens from both sh-NC and sh-circRNA groups. **E** and **F** Expression of miR-142-3p (**E**) and HMGB1 (**F**) by qPCR. n = 3. Data are presented as means ± SD. ***P < 0.001 vs. NC. **G** Whole-body images of mice showing tumor development and metastasis 5 weeks after injection of H1650 cells. (**H** and** I**) The numbers of metastatic foci in lung tissues were caculation according to the HE staining. The data are expressed as the mean ± SD. ***P < 0.001 vs NC

A lentiviral vector was used to deliver sh-circ-YES1 or sh-negative control into H1650 cells, which were subsequently injected into nude mice to induce tumor development. Tumors were measured 5 days after grafting. Compared to the control, circ-YES1 knockdown reduced the xenograft volume and weight (Fig. 5A–C). The immunohistochemistry experiment results revealed decrease in the Ki67 signal, consistent with the inhibition of tumor growth by circ-YES1 knockdown (Fig. 5D). In qPCR analysis, the miR-142-3p level was higher in tumors of mice injected with circ-YES1 siRNA than that in mice injected with negative control siRNA (Fig. 5E); however, the HMGB1 level was lower (Fig. 5F). Imaging of the whole body of mice showed that circ-YES1 knockdown inhibited tumor development and metastasis by decreased the numbers of metastatic foci in lung tissues according to the HE staining (Fig. 5G–I).

## Discussion

The development of NSCLC involves the tumor suppressor gene inactivations and the tumor promoter gene activations (i.e., oncogenes). In the past few decades, several advancements have been made in the early diagnosis and treatment of NSCLC; however, these breakthroughs have not translated into significant improvements in the prognosis of the disease [14]. CircRNAs have important roles in the regulation of several types of cancer [15], and elevated levels of circRNAs have been reported to associate with the clinical manifestations of NSCLC, which include tumor occurrence, stage, and distant metastasis [16, 17]. Here, we report increased circ-YES1 expression in NSCLC. In addition, circ-YES1 knockdown inhibited cell proliferation and migration, and HMGB1 and miR-142-3p were circ-YES1 downstream targets.

CircRNAs can regulate various cellular processes through miRNAs [18]. We discovered that miR-142-3p was a circ-YES1 downstream target. The results of luciferase reporter assays validated the interaction between circ-YES1 and miR-142-3p. Interestingly, a previous study has demonstrated that miR-142-3p could reduce the cancer stem cell phenotypes and the radio-resistance of breast cancer cells [19]. In colorectal cancer, miR-142-3p expression suppressed cell growth through targeting CDK4 [20], indicating that miR-142-3p is a tumor suppressor [21, 22]. In this study, circ-YES1 knockdown increased the miR-142-3p expression level, whereas miR-142-3p inhibition reversed the circ-YES1 knockdown effects on cell migration and proliferation, consistent with the miR-142-3p overexpression experiment results, in which cell migration and proliferation were found to be inhibited.

Here, we report that HMGB1 was a miR-142-3p downstream target. HMGB1, a component protein of chromatin found in all nucleated mammalian cells, was initially discovered in the calf thymus in 1973 [23]. Subsequently, Wang and colleagues demonstrated a role for HMGB1 in sepsis [24], although it has also been reported to participate in the transcriptional regulation of cancer-related genes such as tumor necrosis factor alpha, E-selectin, breast cancer type 1 susceptibility protein, and insulin receptor [25]. HMGB1 overexpression is correlated to poor prognosis of many cancer [26], and HMGB1 is highly expressed in NSCLC cells and tissues [27]. Here, circ-YES1 knockdown and miR-142-3p overexpression decreased HMGB1 expression. Furthermore, HMGB1 or miR-142-3p overexpression reversed the circ-YES1 knockdown effects on cell proliferation and migration, indicating that circ-YES1 can inhibit these processes by regulating the miR-142-3p–HMGB1 axis.

## Conclusion

Circ-YES1 promotes oncogenesis by functioning as an miR-142-3p sponge. Therefore, targeting the circ-YES1–miR-142-3p–HMGB1 axis represents a new approach in the treatment of NSCLC, and our results support the application of circ-YES1 probability as a new prognostic marker in this disease.

## Data Availability

The datasets used and/or analyzed during the current study are available from the corresponding author on reasonable request.

## Abbreviations

circRNAs: Circular RNAs
NSCLC: Non-small cell lung cancer
HMGB1: High mobility group protein B1
