# Correction to: Downregulation of circ-YES1 suppresses NSCLC migration and proliferation through the miR-142-3p–HMGB1 axis

**DOI:** 10.1186/s12931-023-02468-5

**Published:** 2023-07-10

**Authors:** Mingming Jin, Yan Wang, Dawei Zhou, Wanchao Liu, Ruodong Han, Yongbin Chi

**Affiliations:** 1 Shanghai Key Laboratory of Molecular Imaging, Jiading District Central Hospital Affiliated Shanghai University of Medicine and Health Sciences, Shanghai, 201318 China; 2 Department of Clinical Lab, Shanghai Pudong New Area Gongli Hospital, Shanghai Health Commission Key Lab of Artificial Intelligence (AI)-Based Management of Inflammation and Chronic Diseases, Sino-French Cooperative Central Lab, Secondary Military Medical University, , Shanghai, 200135 P.R. China; 3 Department of Ultrasonics, Baoshan District Integrative Medicine Hospital, Shanghai, 201901 China; 4 Department of Clinical Laboratory, Shanghai Baoshan Hospital of Integrated Traditional Chinese and Western Medicine, Shanghai, 201901 P.R. China; 5 Department of Critical Care Medicine, The Affiliated Bozhou Hospital of Anhui Medical University, Bozhou city, Anhui Province 236800 China

**Correction to: Jin et al. Respiratory Research**

10.1186/s12931-023-02378-6

Following publication of the original article [1], the authors identified errors in the author’s affiliation number which tagged in the publication of the article and the unit details of the affiliations. It has been corrected in this correction.

The original article has been corrected.

**Incorrect information:** Mingming Jin1†, Yan Wang5†, Dawei Zhou2†, Wanchao Liu3*, Ruodong Han4* and Yongbin Chi5*

**Correct information:** Mingming Jin^1^†, Yan Wang^2^†, Dawei Zhou^3^†, Wanchao Liu^4^*, Ruodong Han^5^*, Yongbin Chi^2^*

